# Feng-Liao-Chang-Wei-Kang Combined with 5-Fluorouracil Synergistically Suppresses Colitis-Associated Colorectal Cancer via the IL-6/STAT3 Signalling Pathway

**DOI:** 10.1155/2020/1395954

**Published:** 2020-10-03

**Authors:** Lifan Zhong, Fan Yang, Lianfang Gan, Zhaoxin Yang, Shuhong Tian, Mianqing Huang, Chuanzhu Lv, Ling Huang

**Affiliations:** ^1^Hainan Province Key Laboratory for Drug Preclinical Study of Pharmacology and Toxicology Research, Hainan Medical University, Haikou, Hainan, China; ^2^School of Hainan Provincial Drug Safety Evaluation Research Center, Hainan Medical University, Haikou, Hainan, China; ^3^Key Laboratory of Emergency and Trauma of Ministry of Education, Hainan Medical University, Haikou, Hainan, China; ^4^Research Unit of Island Emergency Medicine, Chinese Academy of Medical Science, Hainan Medical University, Haikou, Hainan, China

## Abstract

**Background:**

Colitis-associated colorectal cancer (CAC) develops from active colonic inflammation, which is characterized by the production of proinflammatory cytokines that can induce mutations. IL-6 is produced by multiple cell types located within the tumor microenvironment including tumor-infiltrating immune cells, stromal cells, and the tumor cells themselves. The aim of our study was to explore the mechanism of Feng-Liao-Chang-Wei-Kang (FLCWK) and 5-fluorouracil (5-FU) in treating CAC.

**Method:**

HCT116 cells were treated with 5-FU in the absence or presence of FLCWK. Cell proliferation was assayed by MTT assays. Apoptosis and the cell cycle phases were detected by flow cytometry. Western blotting and Q-PCR assays were used to detect the expression levels of proteins and genes related to the IL-6/STAT3 signalling pathway. A mouse model for CAC was established by treating animals with 12.5 mg/kg azoxymethane (AOM) followed by 3 cycles of 2.5% dextran sodium sulphate (DSS). The associated pathological changes were determined after haematoxylin and eosin (H&E) staining. The expression of related proteins and genes in various tissues was examined using immunofluorescence techniques.

**Results:**

FLCWK enhanced the ability of 5-FU to promote apoptosis by inhibiting the proliferation of HCT116 cells and blocking the IL-6/STAT3 pathway. FLCWK combined with 5-FU reduced the number and size of colon tumors in mice with CAC and significantly increased their survival rate. In the CAC model, FLCWK synergized with 5-FU to inhibit the phosphorylation of STAT3, preventing IL-6/STAT3 signal transduction and thus further inducing apoptosis and inhibition of colon cancer cell proliferation.

**Conclusion:**

FLCWK can inhibit the activation of STAT3 by reducing the production of IL-6, thereby increasing the occurrence of colitis-related colorectal cancer with 5-FU.

## 1. Introduction

Inflammatory bowel disease (IBD) is an umbrella term used to describe a chronic recurring inflammatory state in the intestine [1, 2]. Colitis-associated colorectal cancer (CAC) develops from active colonic inflammation, which is characterized by the production of proinflammatory cytokines that can induce mutations [3]. CAC progresses through the colitis-dysplasia-carcinoma sequence associated with the development of inflammation and indefinite low-grade and high-grade dysplasia that eventually advances to cancer. Among all IBD patients, 10–15% died from colitis-associated colon cancer, with a high mortality rate of about 50% [4].

Multiple cell types located within the tumor microenvironment, including tumor-infiltrating immune and stromal cells and the tumor cells themselves, produce IL-6 [5–8]. When IL-6 binds to its receptor, sIL-6R, it induces dimerization of the gp130 chain and activation of the related Janus kinase (JAK) [9–11]. Phosphorylation of JAK leads to the recruitment and activation of STAT3 [12]. STAT3 is a functional signalling protein, and constitutive activation of STAT3 has been implicated in the progression of inflammation and inflammation-associated cancers [13–16]. Many of the STAT3 target genes [17–20], including the genes encoding Bcl-XL, Survivin, Hsp70, Cyclin D1, c-Myc, etc., have been shown to be upregulated during tumor formation, which may be responsible for mediating CAC [21, 22].

Feng-Liao-Chang-Wei-Kang (FLCWK) granules are a traditional Chinese composite prescription made from the aqueous extract of two medicinal plants, *Daphniphyllum calycinum* Benth. and *Polygonum hydropiper* [23]. *Daphniphyllum calycinum* has been shown to have potent antioxidant and anti-inflammatory properties and contains alkaloids and flavonoids [24]. *Polygonum hydropiper* is known to contain sesquiterpenoids and flavonoids that have antioxidant, anti-inflammatory, and antimicrobial properties [25]. Among them, FLCWK tablets (variety No. ZYB2072002238-1) and FLCWK capsules (variety No. ZYB2072004057) are the national varieties protected by Chinese medicine. Much clinical research and pharmacological experiments have shown that this treatment protects the gastric mucosa, inhibits ulcerative colitis, and has anti-inflammatory analgesic and antibacterial activities [26–28]. The drug 5-fluorouracil (5-FU) is used for the treatment of various solid tumors and is often prescribed to treat digestive tract tumors. However, the use of 5-FU is highly limited [29], as chemoresistance and cytotoxicity occur in cancer and normal cells, and further research is needed to develop more effective alternative or combinational agents for chemopreventive and chemotherapeutic strategies to enhance the effectiveness of 5-FU, while reducing its toxicity and tolerance.

Our previous studies [30, 31] found that prophylactic FLCWK administration significantly inhibited CAC in animal models induced by azoxymethane (AOM)/dextran sodium sulphate (DSS). Moreover, we found that FLCWK significantly inhibited IL-6 and STAT3 mRNA expression in a rat model of ulcerative colitis, which may provide a theoretical pharmacodynamic basis for the enhancement of 5-FU efficacy in CRC by suppressing the IL-6/STAT3 pathway.

In the present study, we used an AOM/DSS mouse model and an *in vitro* colon cancer cell model to evaluate whether FLCWK could increase the antitumor activity of 5-FU. In addition, we examined the effects of this treatment on the IL-6-mediated activity of HCT116 human colon cancer cells, including cell proliferation and apoptosis, STAT3 phosphorylation levels and transcriptional activity, and the multiple target genes of the IL-6/STAT3 signalling pathway.

## 2. Methods

### 2.1. Colon Cancer Cell Culture Techniques

The colonic cancer cell line HCT116 was sourced from the Shanghai Cell Bank of the Chinese Academy of Sciences. Cell lines were grown on in Roswell Park Memorial Institute 1640 (RPMI 1640) containing a supplement of fetal bovine serum (10%) (Gibco Life Technologies, NY, US), in a humidified sterile incubator at 37°C with gaseous CO_2_ (5%) added to the atmosphere.

### 2.2. FLCWK and IL-6 Treatment

FLCWK granules were obtained from Haikou Pharmaceutical Co. Ltd. (Haikou, China). Stock solutions of FLCWK were prepared by dissolving the FLCWK powder in phosphate buffered saline (PBS) to a concentration of 200 mg/mL. The stock solution was diluted to the concentration of the FLCWK working solution in the medium. When cells reached 70–80% confluence, they were pretreated with various concentrations of FLCWK and/or 5-FU (Xudong Haipu Pharmaceutical Co., Ltd., Shanghai, China) in complete 1640 medium for 1 h, followed by stimulation with 40 ng/mL of IL-6 (Sigma Chemical Co., CA, US) for the indicated periods.

### 2.3. Cell Viability Assay

Cells were harvested and resuspended at a final concentration of 1 × 10^5^ cells/mL and seeded in a 96-well plate at 100 *μ*L/well. According to the above method, the cells were incubated for 24 h. Subsequently, 10 *μ*L of a 5 mg/mL solution of 3-(4,5-dimethylthiazol-2-yl)-2,5-diphenyltetrazolium bromide (MTT) (Beyotime, Biotechnology, Shanghai, China) was added to each well and left to react for 4 h. Then, the medium was replaced with dimethyl sulfoxide (DMSO) (150 *μ*L/well) to dissolve the formazan dye and permit reading at an absorbance of 570 nm with an assay reader (Multiskan GO, Thermo Fisher, US).

### 2.4. Analysis of Apoptosis with Flow Cytometry and Annexin V/Propidium Iodide (PI) Staining

After the harvested cells were washed twice with PBS, they were resuspended in binding buffer (500 *μ*L) containing 5 *μ*L of Annexin V-fluorescein isothiocyanate (FITC) (Annexin V-FITC Detection Kit, Beyotime Biotechnology, Shanghai, China). Then, 10 *μ*L of PI was added to the suspension and the cells were incubated for a further 10 min period. The degree of apoptosis was determined using a FACSCalibur cell analyser (Accuri C6, Becton-Dickinson, US).

### 2.5. Cell Cycle Analysis

HCT116 cell cycle progression was determined by flow cytometric analysis using a PI cell cycle assay kit (BD Biosciences, Franklin Lakes, NJ, US). The cells were fixed in 70% ethanol at 4°C overnight. The fixed cells were washed twice with cold PBS and then incubated for 30 min with 0.5 mL of PI/RNase in the dark. The fluorescent signal was detected through the FL2 channel, and the proportion of DNA in various phases was analysed using a FACSCalibur cell analyser.

### 2.6. Western Blot Analysis

Cells were lysed in radioimmunoprecipitation assay (RIPA) buffer (Beyotime Biotechnology, Shanghai, China). A bicinchoninic acid assay (BCA) kit (Beyotime Biotechnology, Shanghai, China) was used to quantify the level of protein in each sample. The protein samples were separated by sodium dodecyl sulphate-polyacrylamide gel electrophoresis and then transferred onto polyvinylidene fluoride (PVDF) membranes (Millipore, MA, US), which were blocked with 5% dried skim milk in TBST for 1 h at 37°C. After appropriate blockade, the PVDF membranes were incubated with the following primary antibodies: STAT3 (1 : 1000), P-STAT3 (1 : 2000) (Cell signalling Technology (CST), MA, US), IL-6 (1 : 1000), IL-1*β* (1 : 1000), CyclinD1 (1 : 200), CDK-4 (1 : 2000), Bcl-2 (1 : 1000), and *β*-actin (1 : 10,000) (all purchased from Abcam, MA, US). The secondary antibodies, goat anti-rabbit IgG (1 : 8000) and goat anti-mouse IgG (1 : 8000), were obtained from Boster Biological Technology Co., Ltd. (Wuhan, China). The blots were incubated with specific antibodies against the indicated primary antibodies overnight at 4°C followed by incubation with secondary antibodies for 1 h at 37°C. Enhanced chemiluminescence chemicals (Biosharp, Seoul, Korea) were employed to examine the target proteins.

### 2.7. Real-Time PCR

Total RNA was extracted from the HCT116 cells or colitis colon tissue with TRIzol reagent (Invitrogen, US), and the quality of the total RNA was assessed by the A260/A280 ratio using a microplate reader (BioTek, US). For real-time quantitative PCR (Q-PCR), reverse transcription was first performed. One microgram of total RNA was reverse transcribed to cDNA using a cDNA Reverse Transcription Kit (TaKaRa, Japan). Then, PCR was performed using SYBR Green PCR Master Mix (Invitrogen, USA). Q-PCR was carried out on an Applied Biosystems Step One Real-Time PCR System (Applied Biosystems, Foster City, CA, US). The relative amount of target mRNA was determined using the comparative threshold (Ct) method by normalizing the target mRNA Ct values to those for GAPDH (ΔCt). The primer sequences were as follows: STAT3:forward:5′TTTGAAGACAGGGACCCTACACAG-3′reverse:5′TCATAGCGGCACATCTCCACA-3′; IL-6:forward:5′-TGTAGTGAGGAACAAGCCAGAG-3′reverse:5′TACATTTGCCGAAGAGCC-3′; IL-1*β*:forward:5′AGGCTGCTCTGGGATTC-3′reverse:5′GCCACAACAACTGACGC-3′; Bcl-2:forward:5′-GACTTCGCCGAGATGTCCAG-3′reverse:5′-GAACTCAAAGAAGGCCACAATC-3′; CyclinD1:forward:5′-GTCCTACTTCAAATGTGTGCAG-3′reverse:5′-GGGATGGTCTCCATCTTAG-3′; GAPDH:forward:5′-CGACCACTTTGTCAAGCTCA-3′ reverse:5′-AGGGGTCTACATGGCAACTG-3′.

### 2.8. STAT3-siRNA Transient Transfection

STAT3 small interfering RNA (RiboBio, Guangzhou, China) was transfected into HCT116 cells using Lipofectamine 2000 (Invitrogen, Carlsbad, CA, US) according to the manufacturer's protocol.

### 2.9. Animal Models

Procedures involving animals were performed in accordance with the Guidelines for the Humane Treatment of Laboratory Animals (Ministry of Science and Technology of the People's Republic of China, Policy No. 2006 398). All experiments were approved by the Institutional Animal Care and Treatment Committee of Hainan Medical University. One hundred and nineteen BALB/c mice (male 5-6 weeks old, weight range: 16–18 g) were purchased from Hunan SJA Laboratory Animal Co., Ltd. (Hunan, China).

**Figure 1:**
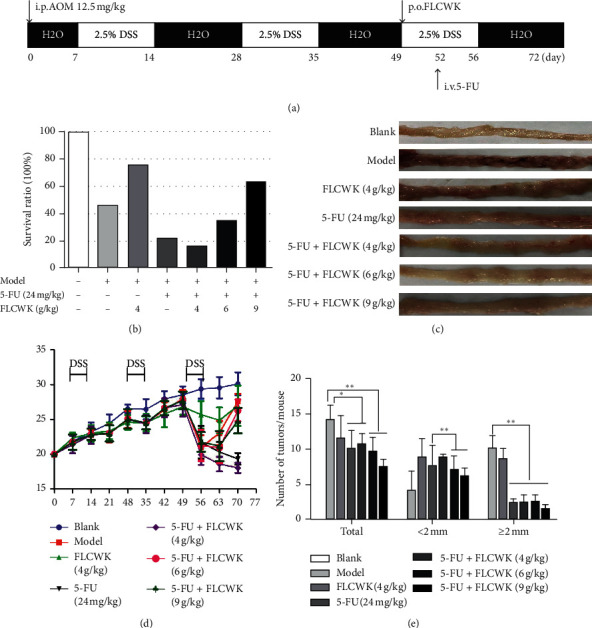
FLCWK administration decreased the occurrence and progression of colonic cancer in AOM/DSS-induced mice (*n* = 6 mice per group). (a) Schematic overview showing the experimental program used for AOM/DSS mice. (b) Mice survival rate. (c) The results of macroscopic observations revealed that FLCWK decreased susceptibility to AOM/DSS-induced colorectal tumor. (d) Basal body weight changes of all groups. FLCWK can increase the body weight in AOM/DSS treated mice. (e) Number of colorectal tumors. Values are mean ± SD of 6 mice. ^*∗*^*P* < 0.05; ^*∗∗*^*P* < 0.01.

The AOM/DSS mouse model has been widely used in the study of carcinogenesis and the development and chemical prevention of colon cancer [32]. All mice were randomly divided into 7 groups, namely, a blank control group (*n* = 17); a model group (*n* = 17); FLCWK (4 g/kg/d, *n* = 17) group; 5-FU (24 mg/kg/d, *n* = 17) group; and the FLCWK (4, 6 and 9 g/kg/d, *n* = 17) combined with 5-FU (24 mg/kg/d) groups. On the first day, for induction of CAC, the mice were intraperitoneally injected with AOM at 12.5 mg/kg. DSS treatment started after AOM administration and lasted for 3 cycles that consisted of 7 days of 2.5% DSS in drinking water, followed by 2 weeks of normal water. The experimental timeline of the animal model is shown in Figure 1(a). All mice were housed in a day and night cycle room (lighting time: 07:30–19:30) and had access to chow *ad libitum*. The body weights of all mice were measured every 2 days, and the activity of the mice was closely observed. At the end of experimental period, mice were anesthetized with ether and killed by cervical dislocation on day 72. On day 72, 0.5 mL blood samples were collected and allowed to settle for 30 min. The blood samples were centrifuged at 3000 rpm for 15 min to obtain the serum. The colon was dissected out immediately, rinsed in ice-cold saline, and then the colon and small intestine were cut longitudinally. The maximum diameter of the tumor was measured using a Vernier caliper, and the number of tumors per mouse was determined. Subsequently, the colon tissue was excised, and the specimens were immediately fixed in formalin or frozen at −80°C for subsequent analysis.

### 2.10. Histopathological Observations

The colon tissue fixed in 10% neutral formalin was dehydrated and embedded in paraffin. Sections of the paraffin-embedded tissues were subjected to haematoxylin and eosin (H&E) staining to evaluate the severity of colitis.

### 2.11. Immunohistochemistry Analysis

Ki-67 (CST, MA, US) was stained by immunohistochemistry. The tissue sections were dewaxed successively from xylene to gradient ethanol solutions. Then, the sections were boiled in citrate buffer solution for 2 min for antigen repair. After the samples were washed with PBS, they were incubated overnight with an antibody at 1 : 100. The sections were incubated with the paired antibodies for 1 h, and then DAB (Zsgb Bio, Beijing, China) staining, haematoxylin staining, and hydrochloric acid differentiation were performed. The sections were incubated with tap water and then successively incubated in ethanol gradients to xylene for dehydration and neutral resin sealing. A brown positive signal was detected under the microscope. Nuclear staining was used as the standard for Ki-67 positive expression.

### 2.12. Immunofluorescence

The protein levels of P-STAT3, IL-6, Bcl-2, and Cyclin D1 in colonic tissue were determined by immunofluorescence. The tissue sections were dewaxed, antigen repair was performed with citrate buffer, and the samples were incubated with the corresponding primary antibody (1 : 100) overnight. Biotin-labelled fluorescent secondary antibody (Boster, Wuhan, China) was incubated for 2 h in the dark, DAPI (Boster, Wuhan, China) was added for 30 min in the dark, and glycerol was used for mounting. The images were observed on a laser confocal fluorescence microscope.

### 2.13. Cytokine Assays

At the end of an experiment, serum was collected and centrifuged for enzyme-linked immunosorbent assays (ELISAs). The concentrations of IL-6, TNF-*α*, and IL-10 were determined by ELISA kits (R&D Systems, Minneapolis, MN, US), according to the manufacturer's instructions.

### 2.14. Statistical Methodology

GraphPad Prism 5.0 was used for most of the statistical analyses. All data are presented as the mean ± standard deviation. SPSS 17.0 software was used to determine the differences between groups via one-way ANOVA, and *P* < 0.05 was considered to be a significant difference.

## 3. Results

### 3.1. FLCWK Enhanced the 5-FU-Mediated Inhibition of HCT116 Cell Proliferation and Promotion of Apoptosis

**Figure 2:**
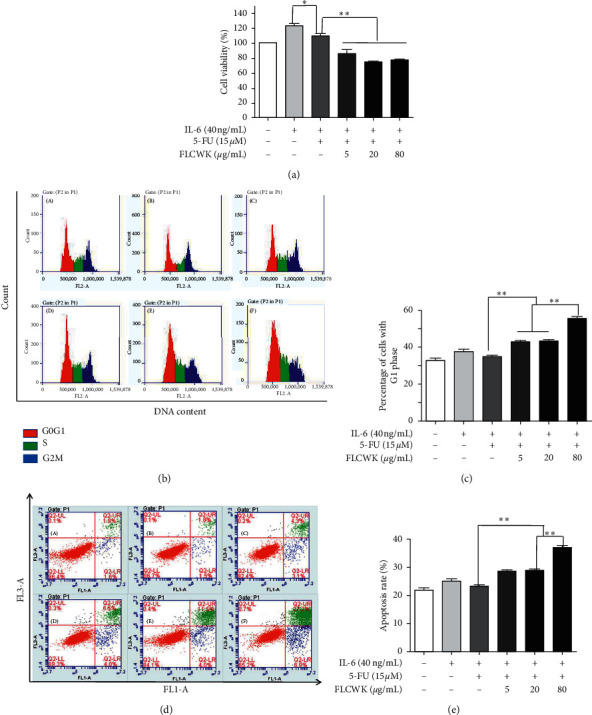
FLCWK- enhanced 5-FU inhibited the viability of HCT116 Cells. Cells were pretreated with various concentrations of FLCWK and/or 5-FU for 1 h followed by stimulation with 40 ng/mL IL-6 for 24 h. (a) Cell viability was determined by MTT assay. (b, c) The proportion of DNA in the GI phase was calculated using a FACSCalibur cell analyser. (d, e) The apoptotic rate was evaluated by flow cytometry via double staining of Annexin V-FITC and PI. Values are mean ± SD of 3 separate experiments. ^*∗*^*P* < 0.05; ^*∗∗*^*P* < 0.01. (A) Blank; (B) IL-6 (40 ng/mL); (C) 5-FU (1 5 *μ*M); (D) 5-FU + FLCWK (5 *μ*g/mL); (E) 5-FU + FLCWK (20 *μ*g/mL); (F) 5-FU + FLCWK (80 *μ*g/mL).

Increased production of IL-6 and enhanced activation of STAT3 have been suggested to be associated with 5-FU resistance. To investigate the effect of IL-6 on the sensitivity to 5-FU in combination with FLCWK, we performed MTT assays. The results confirmed that cell viability changed significantly after different treatments. The activity of the HCT116 cells increased to 112.67% (*P* < 0.05) after stimulation with exogenous IL-6. After 24 h of drug treatment, the cell viability decreased from 109.67% to 74.00% (*P* < 0.01), and the cell inhibitory effect was dose dependent (Figure 2(a)). Furthermore, the effects of the combined treatment on the proliferation and apoptosis of the HCT116 cells were measured using flow cytometry. The G1/S transition is one of the two main checkpoints used by a cell to regulate cell cycle progression and thus cell proliferation [33]. As shown in Figures 2(b) and 2(c), after different treatments, FLCWK and 5-FU produced synergistic inhibition of the proliferation of HCT116 cells by blocking the G1 to S phase transition of the cell cycle. The apoptosis rate of HCT116 cells after IL-6 stimulation was not significantly lower than that of the control group. After administration, the apoptosis rate of HCT116 cells increased significantly, and the apoptosis rate of HCT116 cells treated with 5-FU combined with FLCWK was higher than that of the cells treated with 5-FU alone, an effect that was dose dependent (Figures 2(d) and 2(e)). These results showed that FLCWK could increase 5-FU-induced apoptosis in HCT116 cells.

### 3.2. FLCWK and 5-FU Synergistically Inhibited the IL-6-Activated STAT3 Pathway in HCT116 Cells

**Figure 3:**
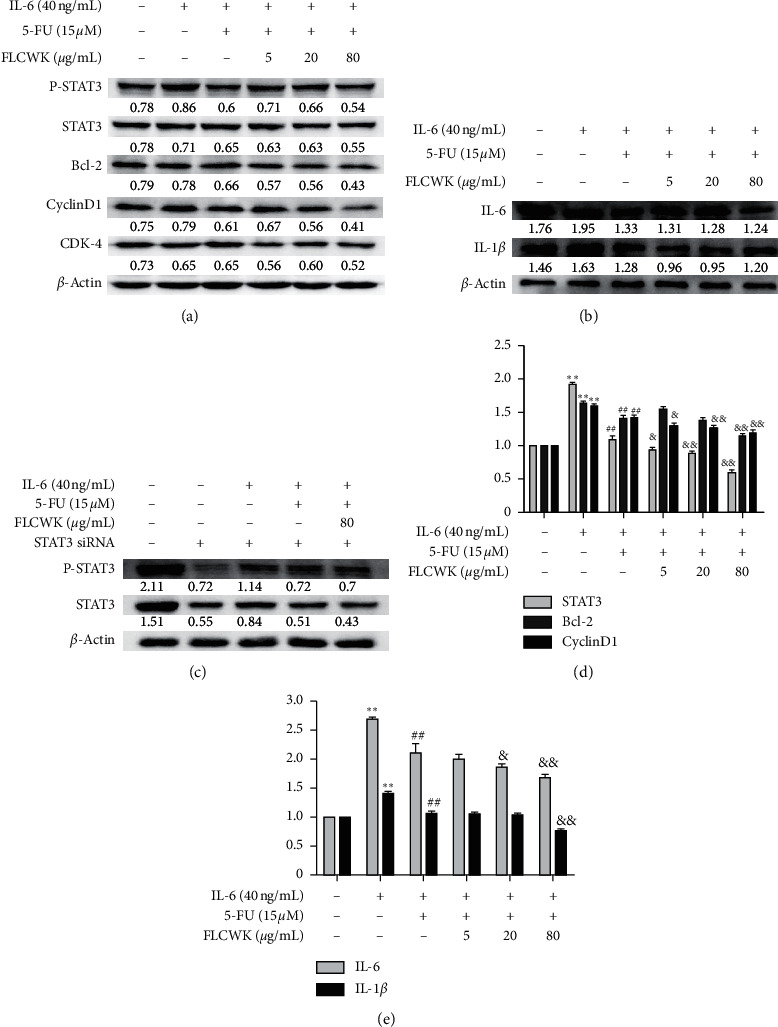
FLCWK-enhanced 5-FU inhibited the IL-6/STAT3 pathway in vitro. HCT116 cells were pretreated with different concentrations of FLCWK and/or 5-FU for 1 h followed by stimulation with 40 ng/mL IL-6 for 24 h. (a) After treatment, total protein was extracted, STAT3 and phosphorylated STAT3, Bcl-2, CyclinD1, CDK4, and *β*-actin were detected by western blot. (b) STAT3 small interfering RNA was transfected into HCT116 cells. The cells were cultured in medium for 72 h after treatment cells were pretreated with various concentrations of FLCWK and/or 5-FU for 1 h followed by stimulation with 40 ng/mL IL-6 for 24 h. Phosphorylated STAT3 was detected by western blot. (c) Total protein of IL-6 and IL-1*β* was detected by western blot (d, e). The messenger RNA levels of STAT3, Bcl-2, CyclinD1, IL-6, and IL-1*β* were measured by Q-PCR. Values are mean ± SD of 3 separate experiments performed. ^*∗*^*P* < 0.05; ^*∗∗*^*P* < 0.01 vs blank group; ^#^*P* < 0.05; ^##^*P* < 0.01 vs IL-6 group; ^&^*P* < 0.05; ^&&^*P* < 0.01 vs 5-FU group.

IL-6/STAT3 signalling plays an important role in the rate of occurrence and development of colon cancer. Abnormal activation of the STAT3 pathway leads to tumor cell apoptosis and an imbalance of cell proliferation [34]. As shown in Figure 3, the mRNA and protein levels of Cyclin D1, CDK4, and Bcl-2 were increased by IL-6 stimulation. The combined 5-FU and FLCWK treatment inhibited the phosphorylation level of STAT3 (P-STAT3) at Tyr705 in a concentration-dependent manner, while the level of nonphosphorylated STAT3 basically remained unchanged. In addition, this treatment induced protein and gene levels of STAT3 downstream proteins, which resulted in the downregulation of mRNA and protein levels of antiapoptotic Bcl-2, Cyclin D1, and CDK4 and the upregulation of the proapoptotic protein Bax. The protein and gene levels of the proinflammatory factors IL-6 and IL-1*β* were also downregulated (Figures 3(c)–3(e)). Figure 3(b) shows that when endogenous STAT3 was knocked down by STAT3 small interfering RNA, the phosphorylated STAT3 level decreased. The level of phosphorylated STAT3 induced by IL-6 also increased, but this phenomenon could be suppressed by 5-FU combined with FLCWK. These results indicated that FLCWK could increase the actions of 5-FU in inhibiting IL-6-induced phosphorylation of STAT3 and its downstream signals.

### 3.3. General Observations of the AOM-DSS Animal Model

The AOM/DSS model can effectively and repeatedly mimic human chronic CAC [32]. This model has been widely used for the study of the carcinogenic mechanisms underlying IBD, especially that of chronic CAC, and the evaluation of drug treatment effects. In the present study, we found that FLCWK was well tolerated by CAC mice and could significantly reduce the toxicity and side effects of 5-FU. The survival rate in the combined group substantially increased with increasing dose compared with those of the model group and the 5-FU alone group (Figure 1(b)). During the third cycle of DSS, the body weight of the CAC mice decreased significantly. After 3 cycles of DSS in the drinking water, the body weight of the mice in the 5-FU single-use group and the low-dose 5-FU combined with FLCWK group did not recover. The mice in the middle and high-dose groups returned to a normal weight. In the FLCWK group, the weight of the mice was always higher than that in the model group (Figure 1(d)). In addition, in the model group, high levels of viscous secretions were observed in the intestinal cavity. The colon mucous membrane in the anus was obviously thickened, the tumors were large and dense, and the number of tumors decreased and dispersed significantly after treatment (Figure 1(c)).  Figure 1(e) shows that the high-dose combined group not only had a significantly lower total number of tumors than the 5-FU alone group but also exhibited significantly fewer tumors >2 mm and <2 mm in size. Therefore, we believe that FLCWK can increase the effect of 5-FU in treating CAC in mice and can reduce the side effects of 5-FU.

**Figure 4:**
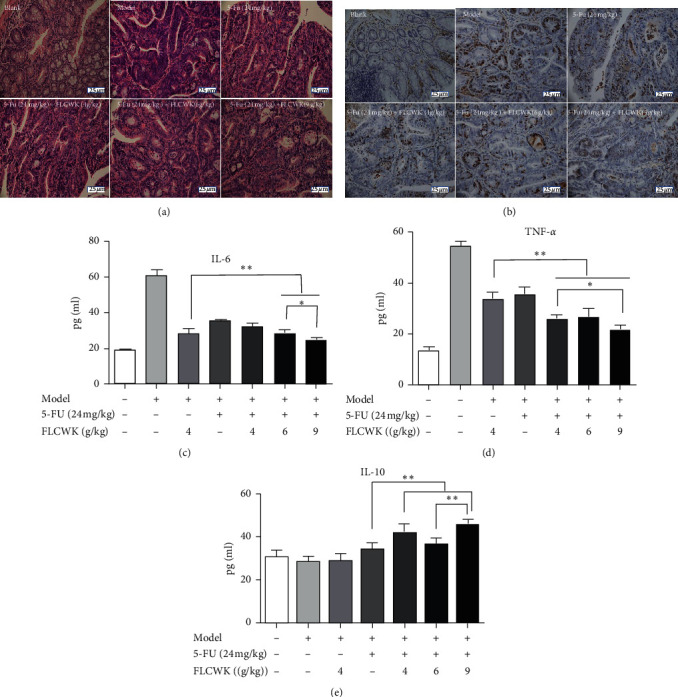
FLCWK can reduce inflammatory response in AOM/DSS-induced mice (*n* = 6 mice per group). (a) H&E-stained sections of colons (×400 magnification). 5-Fu + FLCWK (9 g/kg) group significantly relieved the severe inflammation induced by the AOM/DSS. (b) Expression of Ki-67 was detected by immunohistochemistry; (c–e) Levels of proinflammatory cytokines IL-6, TNF-*α*, and anti-inflammatory cytokine IL-10 in each group. Values are mean ± SD of *n* = 6 mice. ^*∗*^*P* < 0.05; ^*∗∗*^*P* < 0.01.

H&E staining was used to evaluate the incidence of colon cancer and the degree of inflammation in the different groups. The colon tissue structure in the control group was normal, and the glands exhibited an orderly arrangement. In contrast, in the model group, multiple polypoid papillary masses and nodular colon adenocarcinomas were observed, the glands were heteromorphic, and many inflammatory cells were observed in the stroma and muscular layers. Mucosal epithelial cells taken from mice in the treatment group exhibited colonic hyperplasia, nuclear contraction, apoptosis, and enlarged glandular cavities; high levels of shed necrotic tissue were also detected. High-dose FLCWK combined with 5-FU significantly inhibited the extensive infiltration of chronic colitis and inflammatory cells induced by AOM/DSS (Figure 4(a)). These results suggested that FLCWK combined with 5-FU could reduce the incidence of CAC, thus slowing tumor progression in CAC mice.

### 3.4. FLCWK Altered Cytokine Secretion in Colon Tissue

Inflammatory cytokines play an important role in the occurrence and development of colitis. The balance of proinflammatory cytokines and anti-inflammatory factors is essential for the maintenance of human health. The inflammatory cytokines IL-6, IL-10, and TNF-*α* were investigated in these experiments, and serum inflammatory factors were clearly changed after treatment. Figures 4(c)–4(e) show that the levels of the proinflammatory factors IL-6 and TNF-*α* in the model group were significantly higher than those in the control group, while the anti-inflammatory factor IL-10 did not change significantly compared to the control group. The levels of IL-6 and TNF-*α* in the treated group were significantly lower than those in the model group, and the levels of IL-10 were significantly higher than those in the model and control groups. The levels of IL-6 and TNF-*α* in the group which received a high-dose combination of FLCWK were lower than those in the group which was administered 5-FU alone, while the levels of IL-10 were higher than those of the group dosed with 5-FU alone.

### 3.5. Effect of FLCWK Combined with 5-FU on the Proliferation of Tumor Cells in CAC Mice

Changes in cell proliferation can promote tumorigenesis. Immunohistochemical staining was used to observe the effect of Ki-67 expression on CAC cell proliferation. There was an almost linear increase in Ki-67 reactivity from the normal colorectal mucosa through regeneration tissues and various grades of dysplasia to carcinoma [35]. As shown in the Figure 4(b), we observed extended areas of Ki-67 labelled cells in the tumors of the model mice. After co-administration, Ki-67-positive expression was significantly reduced. These findings revealed that FLCWK could synergize with 5-FU to inhibit the proliferation of colon cancer.

### 3.6. FLCWK Inhibited the Proliferation and Apoptosis of Colorectal Tumors by Enhancing the Effects of 5-FU and Repressing IL-6/STAT3 Signalling

**Figure 5:**
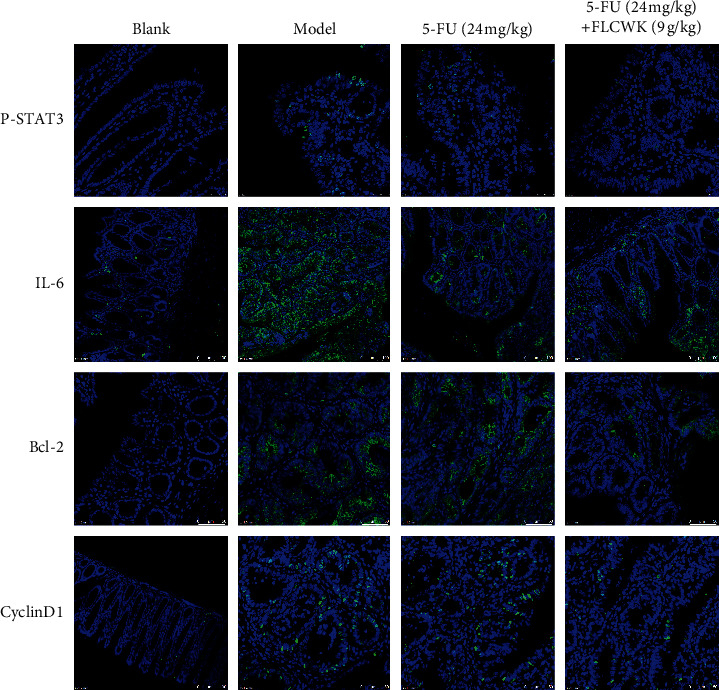
FLCWK-enhanced 5-FU suppressed the development of colitis-associated colon cancer in the AOM-DSS mode. Protein expressions of P-STAT3, IL-6, Bcl-2, and CyclinD1 in the AOM/DSS-treated mice groups were analysed by immunofluorescence.

STAT3 phosphorylation essentially responds to the expression of apoptosis-associated proteins, such as Bcl-2 and Cyclin D1. In human HCT116 cells, we found that FLCWK together with 5-FU could effectively inhibit the IL-6/STAT3 pathway. To confirm further the results of the *in vitro* experiments, we used immunofluorescence to detect the expression of P-STAT 3, IL-6, Bcl-2, and Cyclin D1 in colorectal tumors.  Figure 5 shows that the level of STAT3 phosphorylation in the CAC mice decreased, the expression of the proinflammatory factor IL-6 decreased, and the expression of Bcl-2 and Cyclin D1 also decreased in CAC mice after treatment. The combined treatment was superior to 5-FU alone in inhibiting the infiltration of the inflammatory cells, reducing tumor proliferation and promoting cell apoptosis. The results strongly indicated that FLCWK could increase the phosphorylation of STAT3 with 5-FU, inhibit signal transduction of IL-6/STAT3, and further induce apoptosis and antiproliferative effects in colon cancer cells. We thus concluded that FLCWK could synergize with 5-FU and inhibit suppressed IL-6/STAT3 signalling by attenuating the phosphorylation of STAT3 and further exerting apoptosis-inducing and antiproliferative effects on colon cancer cells.

## 4. Discussion

Chronic inflammation promotes tumor development by inducing gene mutations, inhibiting apoptosis, and stimulating angiogenesis and cell proliferation [36]. The main cause of CAC is mutations and epigenetic changes in the genes related to the regulation of cell proliferation and changes caused by oxidative stress injury induced by chronic inflammatory stimulation. These changes lead to accelerated proliferation and transport of colonic epithelial cells, followed by dysplasia and cancer [37]. Proinflammatory mediators, especially cytokines and chemokines, play a driving role in carcinogenesis and participate in the rate of occurrence and development of cancer [38]. Colon cancer patients are mainly treated with surgery, adjuvant chemotherapy, and radiotherapy. Currently, 5-FU is the main chemotherapeutic drug used to treat colon cancer and gastrointestinal tumors, but its use is limited due to chemotherapeutic resistance and high cytotoxicity [39, 40]. Therefore, the discovery of drugs that will synergize with 5-FU and exhibit high efficacy and low toxicity will improve the chemical sensitivity of colon cancer cells and reduce drug resistance, indicating new much needed strategies to improve the success rate of colon cancer chemotherapy.

FLCWK is commonly used for the treatment of acute and chronic gastroenteritis, IBS, and ulcerative colitis [26–28]. This prescription has significant effects and no adverse reactions. The main components of FLCWK include quercetin, kaempferol, rutin, hyperoside, and other flavonoids. Flavonoids have been reported to induce apoptosis of human cancer cells, inhibit NF-*κ*B and the IL-6/STAT3-mediated inflammatory signalling pathway, and also regulate levels of inflammatory cytokines such as IL-6, IL-1*β*, and TNF-*α* [41, 42].

In the present study, the AOM/DSS mouse model commonly studied to investigate CAC was used to simulate ulcerative colitis-related colon cancer. IL-6 is a major proinflammatory cytokine implicated in the development of CAC [43]. As a proinflammatory mediator, IL-6 binds to gp130 in the tumor microenvironment, resulting in the phosphorylation of STAT3 after JAK activation. STAT3 plays a key role in the regulation of oncogenesis, cell survival, proliferation, angiogenesis, and tumor immunosuppression-related transcription [44, 45]. Our data showed that FLCWK combined with 5-FU could inhibit the phosphorylation of STAT3 in the colon tissues of CAC mice and that FLCWK significantly improved the survival rate of AOM/DSS mice. In addition, this treatment significantly reduced chronic inflammation of the intestine and inhibited the carcinogenic effects caused by AOM/DSS. FLCWK combined with 5-FU significantly reduced the number of tumors, tumor size, and large-scale colorectal adenomas in CAC mice. ELISAs showed that FLCWK combined with 5-FU could effectively reduce the levels of the proinflammatory factors IL-6 and TNF-*α* and promote the production of the anti-inflammatory factor IL-10, thereby reducing the inflammatory response and the activity of the carcinogenic pathway. H&E and immunofluorescence staining revealed that FLCWK combined with 5-FU could reduce the extensive infiltration of inflammatory cells induced by DSS and IL-6 and that phosphorylation of STAT3 was significantly reduced as were the levels of Bcl-2 and CyclinD1 regulated by STAT3. In addition, we demonstrated the effects of FLCWK *in vitro*. FLCWK combined with 5-FU significantly inhibited the IL-6/STAT3 pathway in HCT116 cells. The inhibitory effect of FLCWK combined with 5-FU on CAC may be related to inhibition of cell proliferation, apoptosis, inflammation, and/or the IL-6/STAT3 signalling pathway. In addition to the selective regulation of cytokine signalling, the activation of STAT3 in tumor cells was disturbed, and the increase in proinflammatory cytokines such as IL-6 was further prevented.

## 5. Conclusions

Our research revealed that FLCWK could potentiate the actions of 5-FU and significantly inhibit the development of CAC by regulating the IL-6/STAT3 pathway. Therefore, FLCWK may be a promising tumor chemotherapy adjuvant for the treatment of human CAC.

## Data Availability

All relevant data are included within the article.

## References

[1] Sakurai T., Higashitsuji H., Kashida H. (2017). The oncoprotein gankyrin promotes the development of colitis-associated cancer through activation of STAT3. *Oncotarget*.

[2] Xi M., Wang X., Ge J., Yin D. (2016). *N*′-[(3-[benzyloxy]benzylidene]-3,4,5-trihydroxybenzohydrazide (1) protects mice against colitis induced by dextran sulfate sodium through inhibiting NF*κ*B/IL-6/STAT3 pathway. *Biochemical and Biophysical Research Communications*.

[3] Itzkowitz S. H., Yio X. (2004). Inflammation and cancer IV. Colorectal cancer in inflammatory bowel disease: the role of inflammation. *American Journal of Physiology-Gastrointestinal and Liver Physiology*.

[4] Mattar M. C., Lough D., Pishvaian M. J. (2011). Current management of inflammatory bowel disease and colorectal cancer. *Gastrointestinal Cancer Research*.

[5] Kumari N., Dwarakanath B. S., Das A., Bhatt A. N. (2016). Role of interleukin-6 in cancer progression and therapeutic resistance. *Tumor Biology*.

[6] Nagasak T., Hara M., Nakanishi H. (2014). Interleukin-6 released by colon cancer-associated fibroblasts is critical for tumour angiogenesis: anti-interleukin-6 receptor antibody suppressed angiogenesis and inhibited tumour-stroma interaction. *British Journal of Cancer*.

[7] Bournazou E., Bromberg J. (2013). Targeting the tumor microenvironment. *JAK-STAT*.

[8] Walter M., Liang S., Ghosh S., Hornsby P. J., Li R. (2009). Interleukin 6 secreted from adipose stromal cells promotes migration and invasion of breast cancer cells. *Oncogene*.

[9] Skiniotis G., Boulanger M. J., Garcia K. C., Walz T. (2005). Signaling conformations of the tall cytokine receptor gp130 when in complex with IL-6 and IL-6 receptor. *Nature Structural & Molecular Biology*.

[10] Baran P., Nitz R., Grötzinger J., Scheller J., Garbers C. (2013). Minimal interleukin 6 (IL-6) receptor stalk composition for IL-6 receptor shedding and IL-6 classic signaling. *Journal of Biological Chemistry*.

[11] Haan C., Kreis S., Margue C., Behrmann I. (2006). Jaks and cytokine receptors-an intimate relationship. *Biochemical Pharmacology*.

[12] Cimica V., Chen H. C., Iyer J. K. (2011). Dynamics of the STAT3 transcription factor: nuclear import dependent on Ran and importin-beta1. *PLoS One*.

[13] Lu R., Zhang Y.-g., Sun J. (2017). STAT3 activation in infection and infection-associated cancer. *Molecular and Cellular Endocrinology*.

[14] Yu H., Pardoll D., Jove R. (2009). STATs in cancer inflammation and immunity: a leading role for STAT3. *Nature Reviews Cancer*.

[15] Bromberg J. (2002). Stat proteins and oncogenesis. *Journal of Clinical Investigation*.

[16] Wong A. L. A., Hirpara J. L., Pervaiz S. (2017). Do STAT3 inhibitors have potential in the future for cancer therapy. *Expert Opinion on Investigational Drugs*.

[17] Jiang R., Wang H., Deng L. (2013). IL-22 is related to development of human colon cancer by activation of STAT3. *BMC Cancer*.

[18] Hu B., Zhang K., Li S. (2016). HIC1 attenuates invasion and metastasis by inhibiting the IL-6/STAT3 signalling pathway in human pancreatic cancer. *Cancer Letters*.

[19] Nakamura H., Taguchi A., Kawana K. (2016). STAT3 activity regulates sensitivity to tumor necrosis factor-related apoptosis-inducing ligand-induced apoptosis in cervical cancer cells. *International Journal of Oncology*.

[20] Sinibaldi D., Wharton W., Turkson J., Bowman T., Pledger W. J., Jove R. (2000). Induction of p21WAF1/CIP1 and cyclin D1 expression by the Src oncoprotein in mouse fibroblasts: role of activated STAT3 signaling. *Oncogene*.

[21] Lassmann S., Schuster I., Walch A. (2007). STAT3 mRNA and protein expression in colorectal cancer: effects on STAT3-inducible targets linked to cell survival and proliferation. *Journal of Clinical Pathology*.

[22] Fan Y., Zhang Y.-L., Wu Y. (2008). Inhibition of signal transducer and activator of transcription 3 expression by RNA interference suppresses invasion through inducing anoikis in human colon cancer cells. *World Journal of Gastroenterology*.

[23] Zhang J., Liu X., Fu N., Liu M., Tan Y. (2011). Systemic exposure of quercetin after administration of Feng-Liao-Chang-Wei-Kang granules to rats. *Journal of Ethnopharmacology*.

[24] Gamez E. J. C., Luyengi L., Lee S. K. (1998). Antioxidant flavonoid glycosides fromDaphniphyllumcalycinum1. *Journal of Natural Products*.

[25] Tao J., Wei Y., Hu T. (2016). Flavonoids of *Polygonum hydropiper* L. attenuates lipopolysaccharide-induced inflammatory injury via suppressing phosphorylation in MAPKs pathways. *BMC Complementary and Alternative Medicine*.

[26] Mota K. S., Dias G. E., Pinto M. E. (2009). Flavonoids with gastroprotective activity. *Molecules*.

[27] Gomes A., Fernandes E., Lima J., Mira L., Corvo M. (2008). Molecular mechanisms of anti-inflammatory activity mediated by flavonoids. *Current Medicinal Chemistry*.

[28] Tunon M., Garcia-Mediavilla M., Sanchez-Campos S., Gonzalez-Gallego J. (2009). Potential of flavonoids as anti-inflammatory agents: modulation of pro- inflammatory gene expression and signal transduction pathways. *Current Drug Metabolism*.

[29] Neska J., Swoboda P., Przybyszewska M. (2016). The effect of analogues of 1*α*,25-dihydroxyvitamin D2 on the regrowth and gene expression of human colon cancer cells refractory to 5-fluorouracil. *International Journal of Molecular Sciences*.

[30] Xie Y., Li Y., Huang M. (2018). Assessment of Feng-Liao-Chang-Wei-Kang as a potential inducer of cytochrome P450 3A4 and pregnane X receptors. *Pakistan Journal of Pharmaceutical Sciences*.

[31] Ni P. L., Tian S. H., Yang Z. X. (2019). Feng-Liao-Chang-Wei-Kang is synergistic with 5-fluorouracil in inhibiting proliferation of colorectal cancer. *Asian Pacific Journal of Tropical Medicine*.

[32] Clapper M. L., Cooper H. S., Chang W.-C. L. (2007). Dextran sulfate sodium-induced colitis-associated neoplasia: a promising model for the development of chemopreventive interventions. *Acta Pharmacologica Sinica*.

[33] Wenzel E. S., Singh A. T. K. (2018). Cell-cycle checkpoints and aneuploidy on the path to cancer. *In Vivo*.

[34] Zhang X., Hu F., Li G. (2018). Human colorectal cancer-derived mesenchymal stemcells promote colorectal cancer progression through IL-6/JAK2/STAT3 signaling. *Cell Death & Disease*.

[35] Andersen S. N., Rognum T. O., Bakka A., Clausen O. P. (1998). Ki-67: a useful marker for the evaluation of dysplasia in ulcerative colitis. *Molecular Pathology*.

[36] Kundu J., Surh Y. (2008). Inflammation: gearing the journey to cancer. *Mutation Research/Reviews in Mutation Research*.

[37] Khan I., Ullah N., Zha L. (2019). Alteration of gut microbiota in inflammatory bowel disease (IBD): cause or consequence? IBD treatment targeting the gut microbiome. *Pathogens*.

[38] Lin W.-W., Karin M. (2007). A cytokine-mediated link between innate immunity, inflammation, and cancer. *Journal of Clinical Investigation*.

[39] Bahrami A., Amerizadeh F., Hassanian S. M. (2018). Genetic variants as potential predictive biomarkers in advanced colorectal cancer patients treated with oxaliplatin-based chemotherapy. *Journal of Cellular Physiology*.

[40] Zhang N., Yin Y., Xu S.-J., Chen W.-S. (2008). 5-Fluorouracil: mechanisms of resistance and reversal strategies. *Molecules*.

[41] Wu Y., Fan Q., Lu N. (2010). Breviscapine-induced apoptosis of human hepatocellular carcinoma cell line HepG2 was involved in its antitumor activity. *Phytotherapy Research*.

[42] Lee Y. K., Isham C. R., Kaufman S. H. (2006). Flavopiridol disrupts STAT3/DNA interactions, attenuates STAT3-directed transcription, and combines with the Jak kinase inhibitor AG490 to achieve cytotoxic synergy. *Molecular Cancer Therapeutics*.

[43] Grivennikov S., Karin E., Terzic J. (2009). IL-6 and Stat3 are required for survival of intestinal epithelial cells and development of colitis-associated cancer. *Cancer Cell*.

[44] Park J. H., van Wyk H., McMillan D. C. (2017). Signal transduction and activator of transcription-3 (STAT3) in patients with colorectal cancer: associations with the phenotypic features of the tumor and host. *Clinical Cancer Research*.

[45] Lesina M., Kurkowski M. U., Ludes K. (2011). Stat3/Socs3 activation by IL-6 transsignaling promotes progression of pancreatic intraepithelial neoplasia and development of pancreatic cancer. *Cancer Cell*.

